# FcεRI γ-Chain Negatively Modulates Dectin-1 Responses in Dendritic Cells

**DOI:** 10.3389/fimmu.2017.01424

**Published:** 2017-10-27

**Authors:** Yi-Gen Pan, Yen-Ling Yu, Chi-Chien Lin, Lewis L. Lanier, Ching-Liang Chu

**Affiliations:** ^1^Graduate Institute of Immunology, College of Medicine, National Taiwan University, Taipei, Taiwan; ^2^Institute of Molecular and Genomic Medicine, National Health Research Institutes, Miaoli County, Taiwan; ^3^Institute of Biomedical Sciences, National Chung Hsin University, Taichung, Taiwan; ^4^Department of Microbiology and Immunology, University of California San Francisco, San Francisco, CA, United States; ^5^The Parker Institute for Cancer Immunotherapy, University of California San Francisco, San Francisco, CA, United States

**Keywords:** FcεRI γ-chain, DAP12, dendritic cell, Dectin-1, immunoreceptor tyrosine-based activation motif, SHP-1, PTEN

## Abstract

The inhibitory effect of immunoreceptor tyrosine-based activation motif (ITAM)-containing adapters DAP12 and FcεRI γ-chain (FcRγ) has been found in many immune functions. Herein, we have further explored the role of these adapters in C-type lectin receptors response. We identified that FcRγ, but not DAP12, could negatively regulate the Dectin-1 responses in dendritic cells (DCs). Loss of FcRγ or both DAP12 and FcRγ enhanced the maturation and cytokine production in DCs upon Dectin-1 activation compared to normal cells, whereas DCs lacking only DAP12 showed little changes. In addition, increments of T cell activation and T helper 17 polarization induced by FcRγ-deficient DCs were observed both *in vitro* and *in vivo*. Examining the Dectin-1 signaling, we revealed that the activations of several signaling molecules were augmented in FcRγ-deficient DCs stimulated with Dectin-1 ligands. Furthermore, we demonstrated that the association of phosphatases SHP-1 and PTEN with FcRγ may contribute to the negative regulation of FcRγ in Dectin-1 activation in DCs. These results extend the inhibitory effect of ITAM-containing adapters to Dectin-1 response in immune functions, even though Dectin-1 contains an ITAM-like intracellular domain. According to the role of Dectin-1 in responding to microbes and tumor cells, our finding may have applications in the development of vaccine and cancer therapy.

## Introduction

Dendritic cells (DCs) monitor danger signals in surrounding environment, capture and process antigens, migrate to secondary lymphoid organs, and activate T cells to initiate immune responses (1). DCs are heterogenous but all subsets have intrinsic and cooperative immunoregulatory functions (2). They can direct immune responses either toward to tolerance or inflammatory reactions, which include cellular and humoral immune responses (3, 4). DCs become mature when they encounter pathogen-associated molecules or inflammatory mediators, a key step to determine what function they exert (5). Thus, DCs are provided with diverse receptors that sense microbial components and trigger host responses to invading pathogens. These receptors, known as pattern recognition receptors (PRRs), include toll-like receptors (TLRs), C-type lectin receptors (CLRs), nucleotide-binding oligomerization domain (NOD)-like receptors (NLRs), and RIG-like receptors (RLRs) (6). All PRRs activate specific signaling pathways that lead to expression of genes and tailor immune responses to particular microbes.

CLRs recognize carbohydrate structures present on the pathogens (7). Dectin-1 is a group V CLR possessing a single extracellular carbohydrate-recognition domain (CRD) and a cytoplasmic tail containing an immunoreceptor tyrosine-based activation motif (ITAM)-like domain (8). Dectin-1 is expressed on myeloid cells, including macrophages, DCs, and neutrophils, and is dedicated to the recognition of β-1,3-glucans, which make up to 50% of the fungal cell wall, and is the major receptor for these carbohydrates on leukocytes (9). Upon binding of β-glucan ligands to Dectin-1, the cytoplasmic ITAM-like motif (hemITAM) is able to mediate intracellular signaling and induces a variety of cellular responses. This results in recruitment of spleen tyrosine kinase (Syk) and caspase recruitment domain protein 9 (CARD9), and then induces DC maturation and guides T helper 17 (Th17) differentiation (10). In addition, Src, AKT, PLCγ, and MAPKs are also involved in Dectin-1 signaling pathway. Dectin-1 can recognize a wide variety of fungal species and Dectin-1-deficient mice are susceptible to *Candida albicans* infection, indicating a role for Dectin-1 in antifungal immunity (11). Furthermore, Chiba et al. reported that the recognition of tumor cells by Dectin-1 critically contributes to the antitumor immune responses, implying the applicability of Dectin-1 in cancer therapy (12).

ITAM is critical in signal transduction not only for activation but also inhibition in hematopoietic cells (13, 14). Besides, the ITAM-associated receptors cross talk with other signaling pathways and either augment or dampen these signals (15, 16). Two ITAM-containing adapters, DAP12 (TYROBP) and FcεRI γ-chain (FcRγ), have been identified in myeloid cells and NK cells. DAP12 is a disulfide-bonded homodimer and associates with various activating receptors through its negatively charged aspartic residue present in the transmembrane domain and non-covalently binds to a positively charged amino acid present in the transmembrane of the activating receptor (17). FcRγ is structurally similar to DAP12 but mainly shared by all activating multi-chain Fc receptors (FcRs). FcRs contain a ligand-binding subunit (FcRα) with different extracellular domains, which are the core structure of receptors for IgA (FcαR), IgG (FcγR and FcRn), and IgE (FcεR), and one or two ITAM-containing signaling subunits (FcRγ and FcRβ) shared by multisubunit FcRs (18). Recently, receptors associated with these adapters have been implicated in inhibition of cellular activation, and several models have been proposed (19, 20). In a previous study, we have identified the negative regulation of DAP12 in TLR responses in macrophages (21). Following this finding, we reported that both DAP12 and FcRγ are required for the inhibitory effect on TLR responses in DCs (22). Since DCs are equipped with many types of PRRs, we further examined the regulatory roles of DAP12 and FcRγ in other PRR responses.

Dectin-1 contains a hemITAM for signaling, so the ITAM-containing adapters DAP12 and FcRγ are likely not required for Dectin-1 activation; however, no prior studies have addressed the effect of DAP12 and FcRγ on Dectin-1 response. Here, we explore the involvement of these adapters in Dectin-1 activation by using gene-deficient mice and surprisingly found that FcRγ negatively modulates Dectin-1 responses in DCs.

## Materials and Methods

### Mice and DC/Macrophage Cultures

As previously described (22), bone marrow-derived DCs (BMDCs) were generated from C57BL/6 (National Laboratory Animal Center, Taipei, Taiwan), *Tyrobp*^−/−^, *Fcer1g*^−/−^, and *Tyrobp*^−/−^*Fcer1g*^−/−^ mice. Bone marrow-derived macrophages (BMDMs) were prepared from C57BL/6 and *Fcer1g*^−/−^ mice. In brief, isolated BM cells were cultured in plates with RPMI 1640 medium containing 10% FBS and 10 ng/mL recombinant mouse GM-CSF (315-05, Peprotech) for BMDCs or 10 ng/mL recombinant mouse M-CSF (400-28, Peprotech) for BMDMs. At days 3 and 5, fresh medium containing 10 ng/mL GM-CSF or M-CSF were added into plates. At day 7, non-attached GM-CSF-cultured cells (>80% CD11c^+^ cells) were collected and used for BMDC experiments and attached M-CSF-cultured cells were collected by scraping and used for BMDM experiments.

### DC/Macropgahe Maturation and Cytokine Production

For DC/macrophage maturation, we followed a previously discribed protocol (23) in which cultured DCs/macrophages were treated with curdlan (10 µg/mL, Wako), depleted zymosan (dZym) (10 µg/mL, tlrl-dzn, InvivoGen), or LPS (100 ng/mL, Sigma) at the indicated concentration on day 6. For phosphatase inhibitor treatment, sodium stibogluconate (SS, SHP-1 inhibitor, #567565, Millipore) and SF1670 (PTEN inhibitor, ab141303, Abcam) were incubated for 1 h before Dectin-1 ligand stimulation. After 16 h, the cells were stained with antibodies (Abs) against CD11c (11-0114), CD40 (17-0401), CD80 (12-0801), CD86 (12-0862), and MHC-II (17-5320, all from eBioscience) and analyzed by flow cytometry. For surface expression, 6-day-cultured DCs were stained with Dectin-1 (MCA2289) and Dectin-2 (MCA2415, all from Serotec) for flow cytometry. For cytokine production by DCs, supernatants were collected from BMDC cultures after treatment of curdlan or dZym for 16 h. The secreted cytokines were measured by ELISA kits for IL-2 (88-7024), IL-6 (88-7064), IL-10 (88-7105), and IL-23 (88-7230, all from eBioscience).

### DC Migration

To analyze the migration of DCs, we used chemotaxis assays in transwell chambers as described previously (24). Briefly, BMDCs were treated with curdlan or dZym (10 µg/mL) on day 6, harvested after 16 h, and then seeded in the upper wells of Transwell chambers (pore size, 5 mm; Costar Corning). Culture media without or with CCL21 (200 ng/mL, 250-13, Peprotech) were loaded in the lower wells. After 3 h, the migrated cells in the lower well were collected, stained with anti-CD11c Ab, and then analyzed by flow cytometry.

### Real-time PCR for CCR7 Expression

BM DCs were collected at day 6, treated with dZym (10 µg/mL), and then harvested at the indicated time points. The total RNAs were extracted with TRIzol reagent (Invitrogen) and converted to cDNA by Revert Aid First Strand cDNA Synthesis Kits (Thermo Fisher Scientific) according to the manufacturer’s instructions. SYBR Green real-time PCR was performed with Luminaris Color HiGreen qPCR master mix (Thermo Fisher Scientific) by PikoReal System (Thermo Fisher Scientific). The primers for CCR7 (forward: 5′-AGA GGC TCA AGA CCA TGA CGG A-3′; reverse: 5′-TCC AGG ACT TGG CTT CGC TGT A-3′) and GAPDH (forward: 5′-GAC AAC TTT GGC ATT GTG G-3′; reverse: 5′-ATG CAG GGA TGA TGT TCT G-3′) were used. All mRNA levels of CCR7 were normalized with GAPDH and the fold-change represented the CCR7 expression of dZym-treated BMDCs compared to that of untreated cells.

### Antigen-Specific T Cell Responses

To determine the DC-induced antigen-specific T cell responses, we performed the DC and OT-II T cell coculture *in vitro* (24). Briefly, BMDCs were treated with OVA_323–339_ peptide (10 µg/mL, O1641, Sigma-Aldrich) for 3 h, and then CD4^+^ T cells, which were isolated from OT-II mice by negative selection kits (480005, Biolegend), were added into BMDC cultures at indicated ratio. For proliferation assay, cells were pulsed with [^3^H]thymidine and harvested after 72 h. The proliferation of OT-II T cells was determined by the radioactivity of [^3^H] thymidine incorporation. For detection of IL-17 production, supernatants were collected from DC/OT-II T cell cultures after 4 days. The amounts of IL-17 were measured by ELISA. For recall assay *in vivo*, C57BL/6 and FcRγ-deficient mice were immunized with OVA (50 µg, A2512, Sigma-Aldrich) and incomplete Freund’s adjuvant (AR002, Sigma-Aldrich) mixed with or without curdlan/dZym (200 µg) *via* footpads. After 7 days, inguinal and popliteal lymph node (LN) cells were isolated and the numbers of CD3^+^ T lymphocyte were determined by counting and flow cytometry. For recall response measurement *ex vivo*, total LN cells were seeded in 96-well plate (5 × 10^5^/well) and incubated with OVA at indicated concentrations for 3 days. [^3^H] thymidine was added 12 h before cell harvest. The proliferation of OVA-specific T cells was measured by [^3^H] thymidine incorporation.

### Western Blot

BM DCs were collected from cultures on day 6, washed with PBS, and incubated in serum-free RPMI for starvation. After 3 h, the cells were stimulated with curdlan or dZym (50 µg/mL) and harvested at indicated time points. Then, the cell lysis buffer PhosphoSafe Extraction Reagent (71296, Novagen) was added to the cells and the lysates were resolved by sodium dodecyl sulfate-polyacrylamide gel electrophoresis (SDS-PAGE). When the proteins were transferred from gels to PVDF membranes, the membranes were blocked with PBS containing 5% non-fat milk and the activation of signaling molecules were detected by specific Abs, including anti-phospho (p)-Src (07-909, Millipore), -Src (#2109), -p-Syk (#2701), -Syk (#2712), -p-Akt (Thr308, #9275), -Akt (#9272), -p-PLCγ (#2821), -PLCγ (#2822), -p-ERK (Thr202/Tyr204, #9101, all from Cell Signaling), -ERK (MAB1230, R&D), -p-p38 (Thr180/Tyr182, #9211), -p38 (#9212, all from Cell Signaling), -p-c-Raf (Ser338, #05-538, Millipore), c-Raf (#07-396, Upstate), -FcRγ (06-727, Millipore), -actin (sc-47778, Santa Cruz Biotechnology), -IκBα (#9242, Cell Signaling), and -GAPDH (#MAB374, Millipore). The HRP-conjugated antirabbit IgG (111-035-144) and antimouse IgG (115-035-003, Jackson Immuno Research Laboratories) were used for secondary Abs. The signals were developed by ECL system (Advansta) and exposed on X-ray film (Fuji). Quantification was determined by densitometry using ImageJ software (US National Institutes of Health) and the number represented the fold of each (phosphoprotein/total protein) value normalized to the (phosphoprotein/total protein) value of untreated WT control (WT 0 min).

### Immunoprecipitation

WT BMDCs were collected from cultures on day 6, treated with curdlan or dZym (50 µg/mL), and then harvested at indicated time points. Cells were lysed and the lysates were precleared with protein A/G beads (G-Bioscience) at 4°C for 2 h. After removing the beads, the lysates were incubated with anti-FcRγ Ab (06-727, Millipore) at 4°C for 16 h and then protein A/G beads were added for precipitating Ab-conjugated proteins. After 2 h, the precipitated proteins were resolved by SDS-PAGE and then evaluated by Western blot using anti-SHP-1 (#3759), -SHP-2 (#3397), -SHIP-1 (#2728, all from Cell Signaling), -FcRγ and -PTEN (04-409, Millipore), and -actin Abs. Quantification was determined by densitometry using ImageJ software.

### Statistics

Significances of different Dectin-1 responses between WT and various DAP12- and FcRγ-deficient DCs were determined using a Student’s *t*-test with two-sample equal variance with a two-tailed distribution.

## Results

### Enhancement of Dectin-1-Mediated Maturation in *Fcer1g*^−/−^ DCs as well as *Tyrobp*^−/−^*Fcer1g*^−/−^ DCs

**Figure 1:**
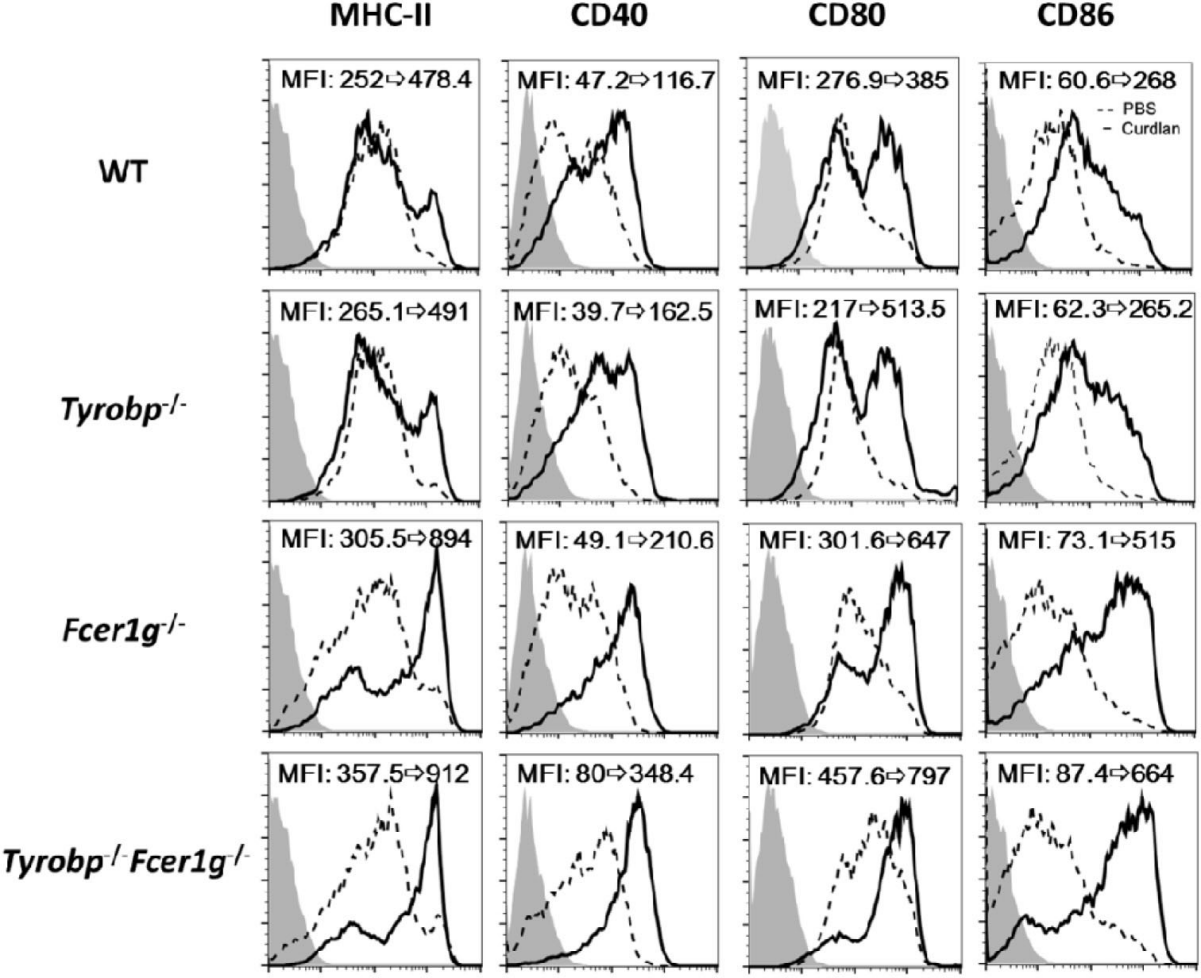
Enhancement of maturation in *Fcer1g*^−/−^ and *Tyrobp*^−/−^*Fcer1g*^−/−^ DCs after Dectin-1 activation. BMDC derived from WT, *Tyrobp*^−/−^, *Fcer1g*^−/−^, and *Tyrobp*^−/−^*Fcer1g*^−/−^ mice were cultured in RPMI containing 10% FBS and 10 ng/mL mGM-CSF, and then treated with PBS (dash line) or curdlan (10 µg/mL, solid line) on day 6. After 16 h, DCs were harvested and the maturation was determined by flow cytometry for the expression of MHC-II, CD40, CD80, and CD86. The changes of mean fluorescent intensities (MFIs) from control to treatment are indicated in each histogram. Gray areas represented the isotype-matched Ig controls. Data shown are representative from three independent experiments.

We have demonstrated the enhanced TLR responses in *Tyrobp*^−/−^*Fcer1g*^−/−^ BMDCs (22). This finding suggests that these ITAM-containing adapters participate in regulating innate immunity of DCs. A notable feature is that TLRs do not need an ITAM for their signaling. In contrast, a typical CLR, Dectin-1, contains a cytoplasmic hemITAM for initiating antifungus activities. Thus, it was interesting to know whether DAP12 and FcRγ have any role in the Dectin-1 responses. To study this hypothesis, we generated BMDCs from WT, *Tyrobp*^−/−^, *Fcer1g*^−/−^, and *Tyrobp*^−/−^*Fcer1g*^−/−^ mice and treated these DCs with a Dectin-1-specific ligand curdlan. Because DC maturation is the key step for DC function, we firstly examined the maturation status of DCs following curdlan stimulation. As shown in Figure 1, the expression levels of MHC class II, CD40, CD80, and CD86 in *Fcer1g*^−/−^ and *Tyrobp*^−/−^*Fcer1g*^−/−^ DCs were clearly higher than that in WT and *Tyrobp*^−/−^ DCs after curdlan treatment. Remarkably, the upregulated levels of maturation were similar between *Fcer1g*^−/−^ and *Tyrobp*^−/−^*Fcer1g*^−/−^ DCs, and the levels of *Tyrobp*^−/−^ DCs were not significantly different from WT DCs (Figure 1). The data indicate a different requirement of these ITAM-containing adapters in Dectin-1 activation compared to TLRs in DCs and suggest that FcRγ, but not DAP12, plays a role in modulating Dectin-1-mediated DC maturation.

### Promoted Cytokine Production by *Fcer1g*^−/−^ DCs as well as *Tyrobp*^−/−^*Fcer1g*^−/−^ DCs after Dectin-1 Activation

**Figure 2:**
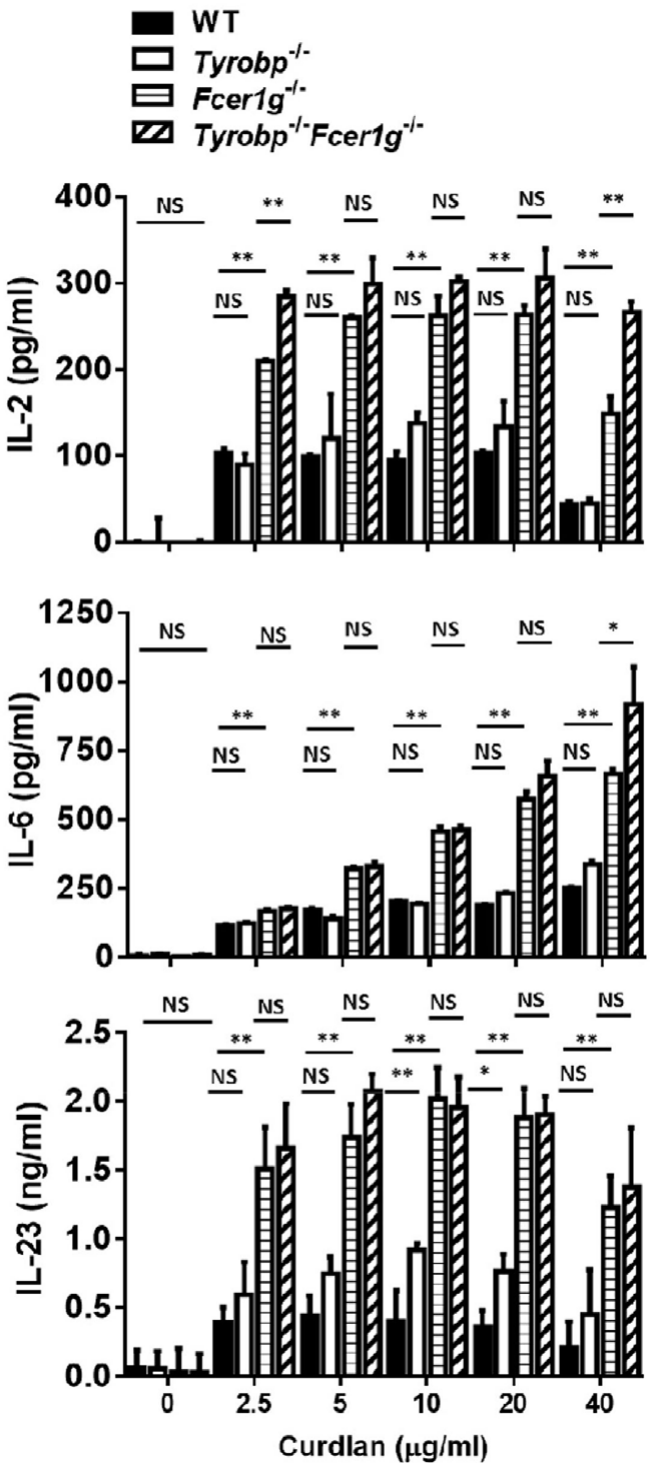
Increase of cytokine production by *Fcer1g*^−/−^ and *Tyrobp*^−/−^*Fcer1g*^−/−^ DCs after Dectin-1 activation. Six-day-cultured BMDCs derived from WT, *Tyrobp*^−/−^, *Fcer1g*^−/−^, and *Tyrobp*^−/−^*Fcer1g*^−/−^ mice were harvested and then incubated with curdlan at indicated concentrations for 16 h. The supernatants were collected and secreted cytokines were measured by IL-2, IL-6, and IL-23 ELISAs. Error bars indicated mean + SD of three independent experiments. The significances *p < 0.05, **p < 0.01, NS, not significant (Student’s *t*-test) were obtained by comparing *Fcer1g*^−/−^ to *Tyrobp*^−/−^*Fcer1g*^−/−^ DCs (upper rows), *Fcer1g*^−/−^ to WT DCs (middle rows), and *Tyrobp*^−/−^ to WT DCs (lower rows) as indicated. Data shown are representative from three separated experiments.

DCs produce various cytokines to induce adaptive immunity after Dectin-1 activation. To investigate whether the lack of DAP12 or FcRγ affects cytokine production by DCs, we measured the amount of various cytokines in WT, *Tyrobp*^−/−^, *Fcer1g*^−/−^, and *Tyrobp*^−/−^*Fcer1g*^−/−^ BMDC cultures by ELISA after curdlan treatment. Similar to what was seen in Dectin-1-induced maturation, *Fcer1g*^−/−^ and *Tyrobp*^−/−^*Fcer1g*^−/−^ DCs had comparable IL-2, IL-6, and IL-23 production and their amounts were significantly higher than that in WT DCs after curdlan treatment. Again, DAP12 deficiency had no significant effect on Dectin-1-mediated cytokine production by DCs (Figure 2). These results show that only FcRγ participates in the negative regulation of cytokine production by DCs after Dectin-1 activation and support the conclusion that FcRγ exclusively inhibits Dectin-1-mediated activation.

### Augmentation of Dectin-1 Responses in *Fcer1g*^−/−^ DCs Was Not due to Quantity and Ligand Specificity of Dectin-1

**Figure 3:**
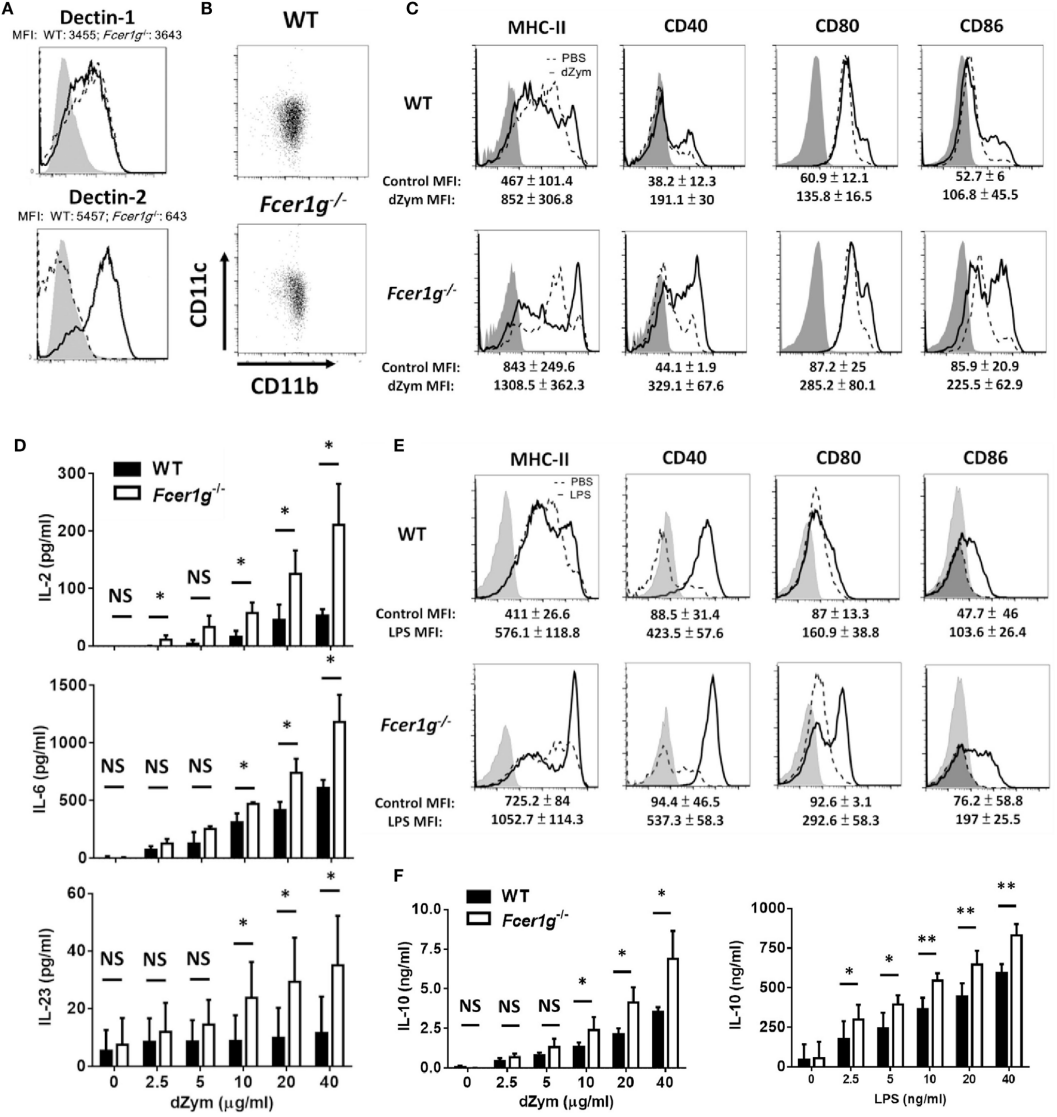
Augmentation of Dectin-1 responses in *Fcer1g*^−/−^ DCs was not due to quantity and ligand specificity of Dectin-1. BMDCs derived from WT and *Fcer1g*^−/−^ mice were cultured for 6 days. **(A)** The expressions of Dectin-1 and Dectin-2 in WT (solid line) and *Fcer1g*^−/−^ (dash line) BMDCs were determined by flow cytometry with gating on CD11c^+^ cells. The MFIs were indicated in each histogram. Gray areas represented the isotype-matched Ig controls. **(B)** The expressions of CD11b and CD11c in WT and *Fcer1g*^−/−^ BMDC cultures were determined by flow cytometry. **(C–F)** For maturation, WT and *Fcer1g*^−/−^ BMDCs were incubated with PBS (dash line), depleted zymosan (dZym) (10 µg/mL, solid line) **(C)**, or LPS (100 ng/mL, solid line) **(E)** for 16 h. The expressions of MHC-II, CD40, CD80, and CD86 were analyzed by flow cytometry. The changes of MFIs (statistic from three independent experiments) from control to treatment were indicated under each histogram. Gray areas represented the isotype controls. All flow data shown are representative from three independent experiments. For cytokine production, WT and *Fcer1g*^−/−^ BMDCs were collected and incubated with dZym or LPS for 16 h. The secreted IL-2, IL-6, and IL-23 by dZym-treated BMDCs **(D)**, and IL-10 by dZym- or LPS-treated BMDCs **(F)** in supernatants were measured by ELISA. Error bars indicated mean + SD of three independent experiments. The significances *p < 0.05, NS, not significant (Student’s *t*-test) were obtained by comparing *Fcer1g*^−/−^ to WT DCs. All data shown are representative from three to five independent experiments.

We have identified the involvement of FcRγ, but not DAP12, in Dectin-1 responses in DCs. Then, we asked whether the augmentation of Dectin-1 responses in *Fcer1g*^−/−^ DCs was determined by external factors. A possibility is that the higher expression of Dectin-1 on the surface of *Fcer1g*^−/−^ DCs results in the increment of its responses. To test this hypothesis, we measured the surface expression of Dectin-1 in WT and *Fcer1g*^−/−^ DCs by flow cytometry. As shown in Figure 3A, the expression level of Dectin-1 on *Fcer1g*^−/−^ DCs was comparable to that on WT cells, excluding a quantitative effect of Dectin-1. Because FcRγ is required for the surface expression of Dectin-2, we simultaneously compared the Dectin-2 expression and confirmed the FcRγ deficiency in *Fcer1g*^−/−^ DCs through the loss of Dectin-2 expression (Figure 3A). In addition, we also affirmed that FcRγ deficiency did not affect the DC phenotype *in vitro* as CD11b and CD11c expressions were similar between WT and FcRγ-deficient BMDCs (Figure 3B).

Next, we used another Dectin-1 ligand dZym to check whether the enhanced phenotypes of *Fcer1g*^−/−^ DCs are ligand-specific. Consistent with the trend seen with curdlan stimulation, maturation (Figure 3C) and cytokine production (Figure 3D) were increased to a greater extent in the *Fcer1g*^−/−^ DCs in comparison with WT DCs. We performed LPS stimulation as another quality control of DCs and also displayed the enhancing effects of FcRγ deficiency on LPS-induced DC maturation (Figure 3E) and IL-10 production (Figure 3F). Collectively, these results imply an intracellular modulation of FcRγ on the quality of Dectin-1 activation in DCs.

**Figure 4:**
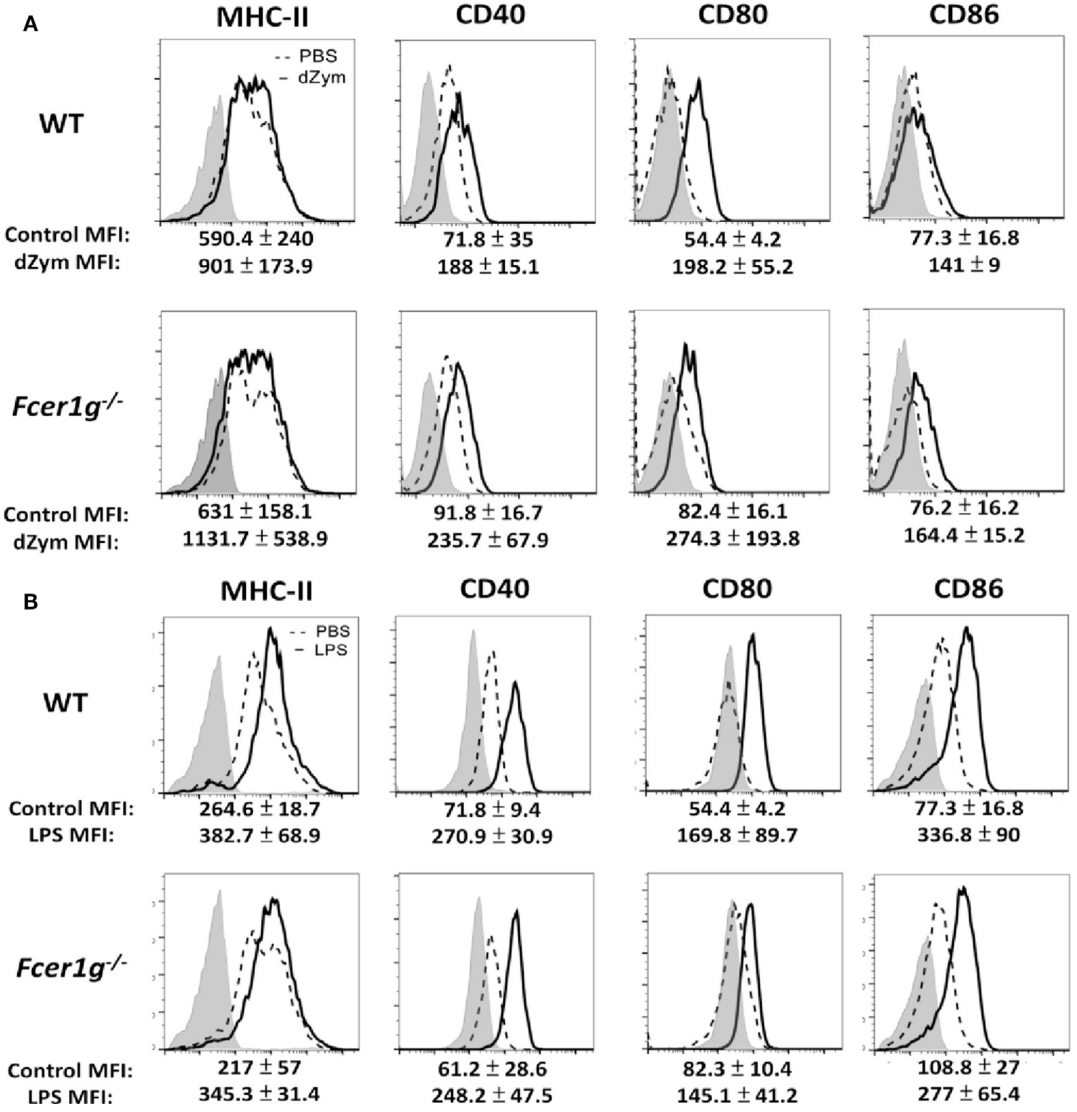
Loss of FcεRI γ-Chain did not affect the maturation of macrophages. Bone marrow-derived macrophages derived from WT and *Fcer1g*^−/−^ mice were cultured for 6 days. Cells were then incubated with PBS (dash line), dZym (10 µg/mL, solid line) **(A)**, or LPS (100 ng/mL, solid line) **(B)** for 16 h. The expressions of MHC-II, CD40, CD80, and CD86 were analyzed by flow cytometry. The changes of MFIs (statistic from three independent experiments) from control to treatment were indicated under each histogram. Gray areas represented the isotype controls. All flow data shown are representative from three independent experiments.

We have confirmed the negative regulation of FcRγ deficiency in DCs. It is interesting to know whether macrophages have the same phenotype. After stimulation, the Dectin-1- (Figure 4A) and LPS- (Figure 4B) induced maturation has no significant increase in FcRγ-deficient macrophages compared to WT cells, demonstrating that FcRγ may be not involved in modulating Dectin-1 responses in macrophages.

### Loss of FcRγ Had No Effect on the Migration of Dectin-1-Activated DCs

**Figure 5:**
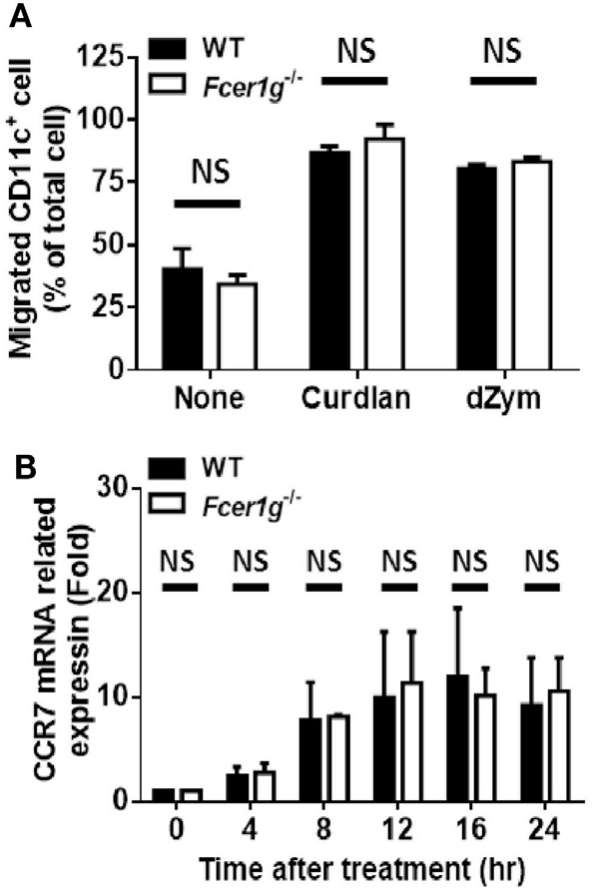
Loss of FcεRI γ-chain did not change the migration of Dectin-1-activated DCs. BMDCs derived from WT and *Fcer1g*^−/−^ mice were cultured for 6 days. **(A)** WT and *Fcer1g*^−/−^ BMDC cultures were treated with curdlan or dZym for 16 h. Cells were harvested and transferred to upper wells of Transwell chambers, and then CCL21 (200 ng/mL)-containing media were loaded into lower wells. After 3 h, the numbers of CD11c^+^ cell in lower wells were determined by counting and flow cytometry. **(B)** WT and *Fcer1g*^−/−^ BMDCs were harvested and treated with dZym for the indicated times. Then, total RNAs were extracted for analyzing CCR7 mRNA level by real-time PCR. The significances NS, not significant (Student’s *t*-test) were obtained by comparing *Fcer1g*^−/−^ to WT DCs. Error bars indicated mean + SD of three independent experiments. All data shown are representative from three independent experiments.

Activated DCs upregulate CCR7 expression and migrate to CCL19/21-expressing draining LNs to initiate adaptive immune responses. Therefore, we studied the effect of FcRγ deficiency on the ability of DC migration after Dectin-1 activation by chemotaxis assays using transwell chambers. Unexpectedly, the CCL21-guiding migration of *Fcer1g*^−/−^ DCs was not different from that of WT cells (Figure 5A). Because CCR7 is the receptor of CCL21 on DCs, we measured the CCR7 expression of DCs after dZym stimulation. Consistently, the mRNA levels of CCR7 in *Fcer1g*^−/−^ DCs were similar to that in normal cells (Figure 5B). Thus, the FcRγ deficiency does not affect the CCR7 expression and migration of Dectin-1-activated DCs.

### Antigen-Specific T Cell Responses Were Increased in *Fcer1g*^−/−^ Mice

**Figure 6:**
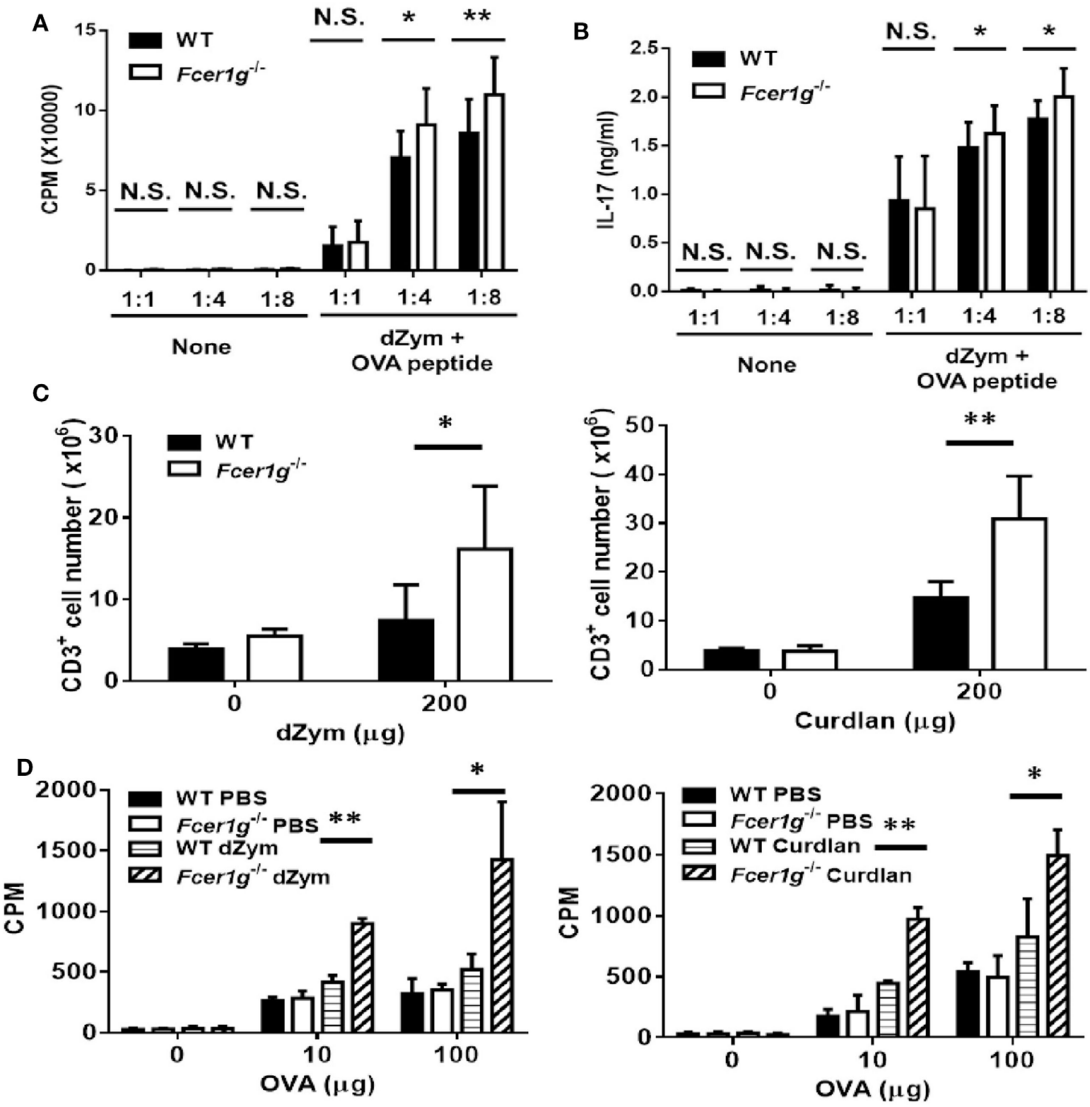
OVA-specific T cell responses were promoted in *Fcer1g*^−/−^ mice. **(A,B)** Six-day-cultured BMDCs derived from WT and *Fcer1g*^−/−^ mice were pulsed with OVA323-339 peptide for 3 h, then CD4^+^ OT-II T cells isolated from OT-II transgenic mice were cultured with these BMDCs at indicated ratios for 3 days. The proliferation of T cells was determined by [^3^H]thymidine incorporation **(A)** and IL-17 production was detected by ELISA **(B)**. **(C,D)** WT and *Fcer1g*^−/−^ mice were immunized with OVA (50 µg) mixed with incomplete Freund’s adjuvant and dZym (left panel) or curdlan (right panel) *via* footpads. After 7 days, the draining lymph nodes were isolated and total cells were collected. **(C)** The numbers of CD3^+^ cell were determined by counting and flow cytometry. **(D)** Total cells were cultured in 96-well plates with the indicated amounts of OVA for 3 days. The proliferation of T cells was measured by [^3^H]thymidine incorporation. Error bars indicated mean + SD of three independent experiments. The significances *p < 0.05, **p < 0.01 (Student’s *t*-test) were obtained by comparing *Fcer1g*^−/−^ to WT dendritic cells. All data shown are representative from three independent experiments.

Following migration, the mature DCs induce antigen-specific T cell activation and proliferation in draining LNs. Thus, we determined the responses of antigen-specific T cell population *in vitro* and *ex vivo*. First, WT and *Fcer1g*^−/−^ BMDC were cultured with OT-II T cells at different ratio and the proliferation of OT-II T cells were detected by [^3^H]thymidine incorporation after 3 days. As shown in Figure 6A, *Fcer1g*^−/−^ BMDC could significantly induce more T cell proliferation than WT BMDC. In addition, higher production of IL-17 was detected in *Fcer1g*^−/−^ BMDC/T cell cultures (Figure 6B). To further confirm the phenotype *ex vivo*, we did antigen-specific recall assay on WT and *Fcer1g*^−/−^ mice to compare T cell proliferation. WT and *Fcer1g*^−/−^ mice were immunized with OVA mixed with incomplete Freund’s Adjuvant plus curdlan or dZym *via* foot pads. After 7 days, draining LNs were collected and the number of T cell was calculated. Significantly, the size (data not shown) and number of T cells in draining LNs of *Fcer1g*^−/−^ mice were greater than that in WT mice after immunization (Figure 6C). Furthermore, we measured OVA-specific T cell proliferation by adding OVA into the cultures of isolated LN cells for 72 h and counting incorporated [^3^H] thymidine. As shown in Figure 6D, the proliferation of OVA-specific T cells in *Fcer1g*^−/−^ LN cells was more intense than that in WT cells. The elevated activation of OVA-specific T cells in immunized *Fcer1g*^−/−^ mice could be due to the higher expression levels of MHC class II and costimulators in Dectin-1-activated *Fcer1g*^−/−^ DCs (Figures 1 and 3C), and supported the conclusion that loss of FcRγ causes a relative promotion in DC activation by Dectin-1 stimulation.

### Enhanced Dectin-1 Signaling in *Fcer1g*^−/−^ DCs

**Figure 7:**
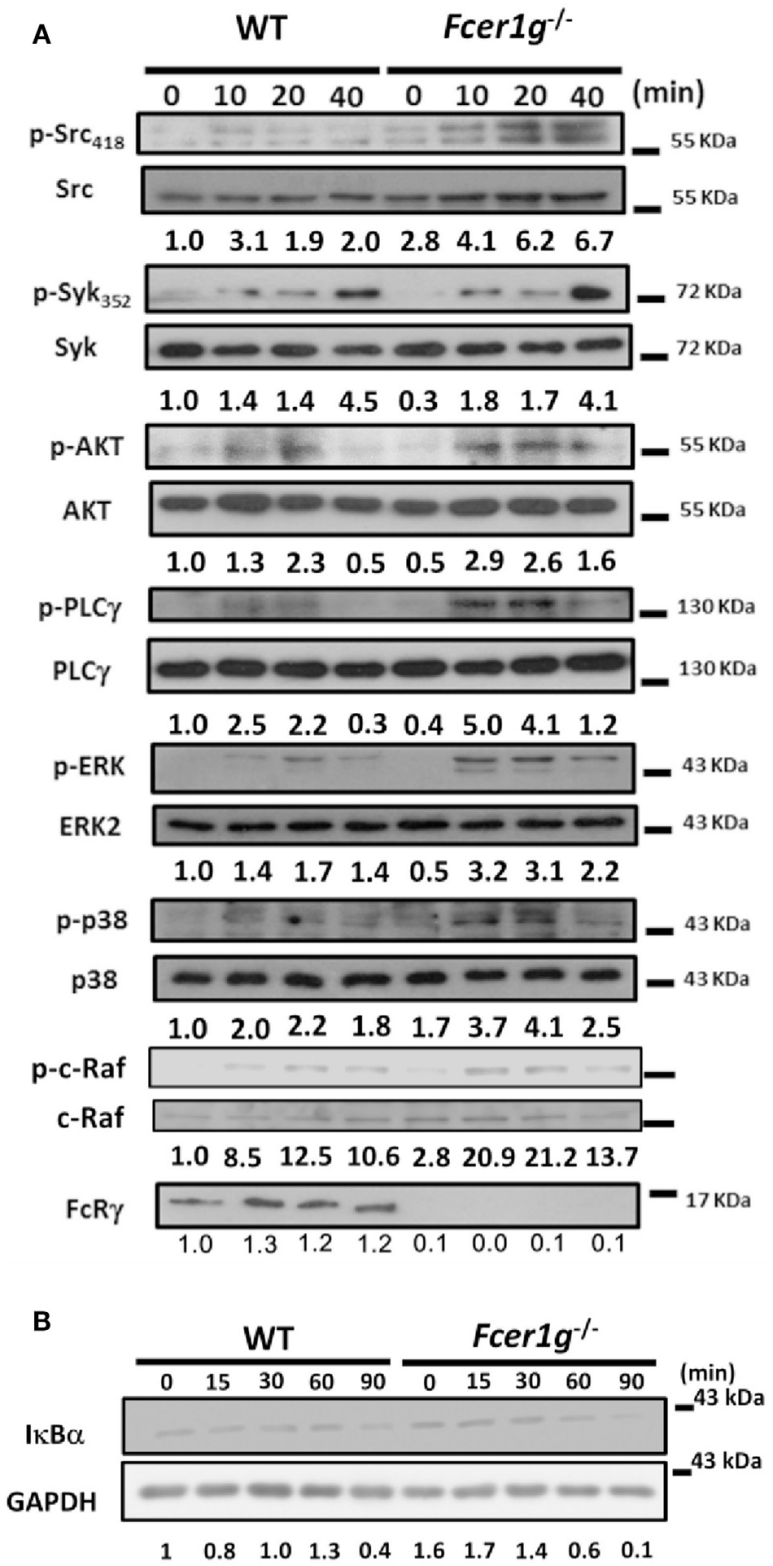
Enhanced Dectin-1 Signaling in *Fcer1g*^−/−^ DCs. Six-day-cultured BMDCs derived from WT and *Fcer1g*^−/−^ mice were collected and starved in serum-free RPMI. After 3 h, cells were treated with dZym and lysed at indicated time points. The proteins were separated by SDS-PAGE, transferred to PVDF membranes, and then analyzed by Western blot. The detection of phosphorylated and total proteins of Src, Syk, AKT, PLCγ, ERK, p38MAPK, and c-Raf **(A)**, and total proteins of IκBα and GAPDH **(B)**, were shown. Quantification was determined by densitometry using ImageJ software and the number represented the fold of each (phosphoprotein/total protein) value normalized to the (phosphoprotein/total protein) value of untreated WT control (WT 0 min). All data shown were representative from three to five independent experiments.

We have confirmed the intracellular effect of FcRγ on Dectin-1 response in DCs (Figure 3A). To explore the mechanism, we examined the signaling events of Dectin-1 in DCs after Dectin-1 activation. According to previous research, Dectin-1 ligands treatment can induce both Syk-dependent and Syk-independent signaling activation. Thus, we compared the activation status of several molecules in both Dectin-1-mediated signaling between WT and *Fcer1g*^−/−^ DCs by Western blot. The phosphorylation levels of Src, AKT, PLCγ, ERK, p38MAPK, and c-Raf were increased in *Fcer1g*^−/−^ DCs compared to WT cells (Figure 7A). Moreover, IκB degradations were accelerated in *Fcer1g*^−/−^ DCs compared to WT cells, represented the enhancement of NF-κB activation (Figure 7B). The blotting data strongly suggest that FcRγ suppresses the Dectin-1 signaling and then reduces the Dectin-1 responses in DCs. However, the phosphorylation levels of Syk were not significantly different between WT and *Fcer1g*^−/−^ DCs (Figure 7A).

### Recruitment of Phosphatases by FcRγ after Dectin-1 Activation

**Figure 8:**
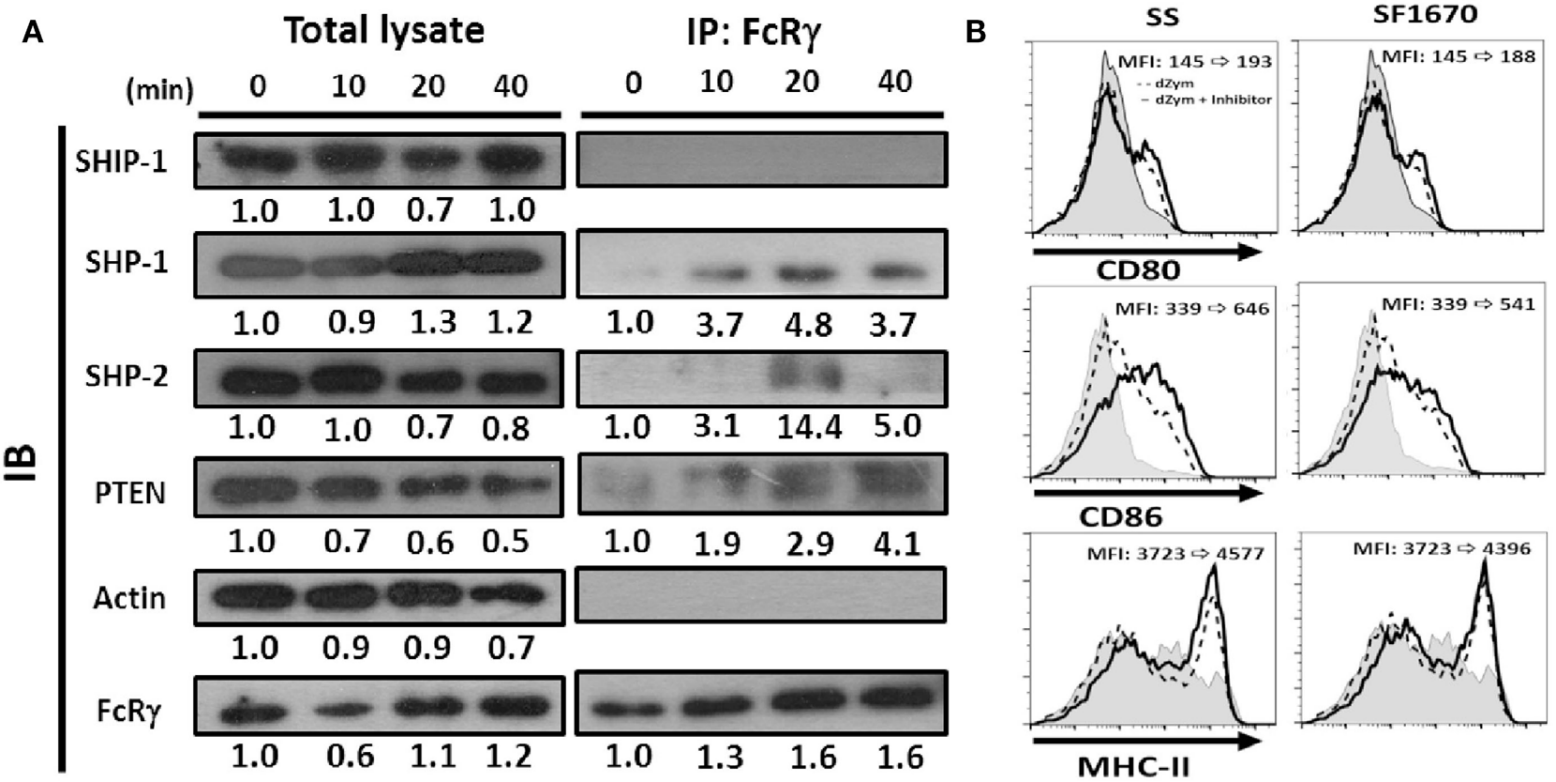
Phosphatases were recruited by FcRγ and negatively regulated Dectin-1 responses in DCs. Six-day-cultured BMDCs derived from WT mice were collected and treated with dZym. **(A)** Cell lysates were collected at indicated time points and incubated with anti-FcRγ Ab. After 16 h, protein A/G beads were added for precipitation. The phosphatases SHIP-1, SHP-1, SHP-2, and PTEN in whole cell lysate or associated proteins with FcRγ were detected by SDS-PAGE and Western blot. Quantification was determined by densitometry using ImageJ software. **(B)** BMDCs were incubated with sodium stibogluconate (SS) (SHP-1 inhibitor) or SF1670 (PTEN inhibitor) for 1 h before dZym treatment. After 16 h, the expressions of CD80, CD86, and MHC-II were detected by flow cytometry. The changes of MFIs from control to treatment are indicated in each histogram. Gray areas represented the isotype-matched Ig controls. All data shown were representative from three to five independent experiments.

The ITAM domain in the FcRγ has been shown to bind Src homology 2 (SH2)-containing protein tyrosine phosphatases (PTPs) and inhibit cell activation (25, 26). Thus, we examined whether any SH2-containing PTP was recruited to FcRγ after Dectin-1 activation in DCs. FcRγ was immunoprecipitated after Dectin-1 ligand treatment, and then the possible PTPs, including SH2 domain-containing phosphatase-1 (SHP-1), SHP-2, SH2 domain-containing inositol 5′-phosphatase-1 (SHIP-1), were determined by Western blot. As shown in Figure 8A, SHP-1 and SHP-2 were present in the FcRγ immunoprecipitate. However, SHIP-1 was absent. Because the enhanced AKT activation was observed in *Fcer1g*^−/−^ DCs after Dectin-1 activation (Figure 7A), we were guided to examine the phosphoinositide phosphatase PTEN (phosphatase and tensin homolog), which is a suppressor for AKT activation (27), and surprisingly found the presence of PTEN in FcRγ immunoprecipitate (Figure 8A). The results illustrate that SHP-1, SHP-2, and PTEN are recruited to FcRγ after Dectin-1 activation and probably involved in the negative modulation of FcRγ to Dectin-1 responses in DCs.

In order to support the potential functional implication of these phosphatases in the suppressive effects, we treated WT DCs with chemical inhibitors to examine whether the inhibitory effect could be reverted. It has been reported that SHP-2 can positively regulate Dectin-1-induced activation in response to fungal infection (28). Thus, we focused on SHP-1 and PTEN. As shown in Figure 8B, when we used specific inhibitors SS (for SHP-1) and SF1670 (for PTEN) for blocking phosphatase activities, both SS- and SF1670-treated BMDCs expressed higher level of CD80, CD86 and MHC-II after dZym treatment. These results confirmed that SHP-1 and PTEN could be involved in the negative regulation of FcRγ in Dectin-1 responses.

## Discussion

The inhibitory effects of ITAM-containing adapters DAP12 and FcRγ have been elucidated in myeloid cells (13). In a previous study, we identified that both DAP12 and FcRγ are required for inhibition of TLR responses in DCs (22). Because ITAM is not involved in TLR signaling, we then examined the effect of DAP12 and FcRγ on Dectin-1, which contains a hemITAM, in this study. We surprisingly found that *Fcer1g*^−/−^ and *Tyrobp*^−/−^*Fcer1g*^−/−^ DCs, but not *Tyrobp*^−/−^ DCs, showed similar hyper-responsive phenotypes, suggesting that only FcRγ plays a role in negatively regulating Dectin-1 responses in DCs. Consistently, we have observed that FcRγ most likely plays a more important role than DAP12 in some TLR activities in DCs (22). Although some activation markers already increased basally in the absence of FcRγ (Figures 1 and 3C,D), probably due to another ligand with another signaling pathway that is present in those culture conditions, the enhancement of Dectin-1 responses was really significant in *Fcer1g*^−/−^ DCs both *in vitro* and *in vivo*. In contrast, the Dectin-1 responses of WT and *Fcer1g*^−/−^ BM-derived macrophages were not different (Figure 4). Although the requirement of DAP12 and FcRγ for negative regulation is probably dependent on the types of cell or receptor, it seems that FcRγ is more influential than DAP12 in DCs, comparing to macrophages in which DAP12 is more important than FcRγ (21). A possibility is that the profiles of DAP12- and FcRγ-associated receptors recruited by TLR and Dectin-1 ligands are different between DC and macrophage. Further studies are required to identify the “co-receptors” in these myeloid cells.

The ITAM has been characterized in many immune receptors for initiating signal transduction. However, increasing evidences establish its negative role in immune responses. Our group first reported enhanced TLR responses in DAP12-deficient macrophages (21), and both DAP12 and FcRγ are required for the enhancement of TLR responses in DCs (22). Subsequently, the DAP12-associated triggering receptor expressed on myeloid cell-2 (TREM-2) was also reported to negatively regulate TLR responses in DCs (29). In addition, ITAM-associated receptors inhibit type I IFN (IFN-α/β) signaling in primary human macrophages (30). Suppression by ITAMs has also been found in lymphocytes. CD79a and CD79b ITAMs mediate an inhibitory signaling cascade required for B cell anergy (31). *In vivo*, Gmyrek et al. show that mice lacking both DAP12 and FcRγ possess a greatly enhanced monocyte-derived DC differentiation, IL-12 production, and CD8 T cell responses (32). A well-characterized function of ITAM is the inhibitory effect of the IgA receptor FcαRI. Monomeric IgA binds to FcαRI and results in partial phosphorylation of its FcRγ, which then attenuates immune activation (20). Our study identifies a new target modulated by ITAM in immune responses.

An interesting question is how the FcRγ affects the “non-adapter-associated receptor” Dectin-1 in DCs. Bezbradica and Medzhitov have suggested that many immunoreceptors, all of which use a conserved ITAM-module for their signaling, can couple with members of additional classes of membrane receptors to deliver unique signal(s) to the cell (16). In studies of FcRγ, many reports have documented the inhibitory effects of FcRγ-associated FcRs. Monovalent targeting of FcαRI triggers inhibitory ITAM (ITAMi) signaling through the associated FcR γ chain (33). In addition, FcγRI engaged with immune complexes reduces the production of IFN-β and TLR4 signaling and increases the secretion of IL-10 (34). Another FcR, FcγRIIA, can ameliorate inflammation by engagement with anti-FcγRII F(ab′)2 fragments or intravenous hIgG (IVIg) treatment (35). Recently, the macrophage inducible C-type lectin (Mincle) has been found to form a complex with macrophage C-type lectin and FcRγ (36) and is a fungal receptor that can suppress Dectin-1-induced antifungal immunity (37) and *Fonsecaea pedrosoi*-induced Th17-cell differentiation in mice (38). Mincle can also be shifted to an ITAMi configuration by Leishmania and then impairs DC activation (39). Thus, we proposed a model that an FcRγ-associated receptor might couple with Dectin-1 after curdlan or dZym treatment, and then SHP-1 and PTEN were recruited by FcRγ to modulate Dectin-1 signaling. However, we did not know what the Dectin-1-coupled FcRγ-associated receptor is. Future studies would explore whether Mincle, a possible candidate, could be recruited to Dectin-1 after curdlan/dZym treatment in DCs in the future.

We have confirmed that the inhibitory effect of FcRγ is due to modulation of Dectin-1 signaling but not reduction of Dectin-1 expression. The activities of several molecules in Dectin-1 signaling were enhanced in FcRγ-deficient DCs, including Src, AKT, PLCγ, ERK, p38MAPK, c-Raf, and NF-κB (Figure 7). Interestingly, the FcRγ deficiency did not significantly affect the Syk activation (Figure 7A). It has been shown that Syk is required for Mincle-FcRγ-dependent inhibitory axis during Leishmania infection (39), implying that Syk could also be activated by FcRγ-mediated suppression after Dectin-1 stimulation. Although Syk activation was not increased in FcRγ-deficient DCs, probably due to the saturated activity after curdlan/dZym treatment, the entire activation might only trigger Dectin-1 signaling without shared by FcRγ-mediated negative signaling and resulted in the enhancement of Dectin-1 downstream signaling. In addition to these signal molecules, the transcription factor IRF5-mediated Dectin-1-induced IFN-β production by renal DCs is crucial for defense against *C. albicans* infection (40). However, we did not see the effect of FcRγ deficiency on IRF5 activation and IFN-β production after Dectin-1 activation (data not shown).

What is the mechanism for suppressing Dectin-1 activation by FcRγ in DCs? It has been shown that low avidity ligation of FcαRI induces the translocation of FcαRI associated with SHP-1 to membrane lipid rafts. Subsequent ligation of activating receptors results in their colocalization with FcαRI and SHP-1, and then SHP-1 exerts cell inhibition of multiple types of activation signals (25). A recent study also described that Leishmania triggers a Mincle-dependent inhibitory axis characterized by SHP-1 coupling to the FcRγ chain (39). We did observe the association of SHP-1 with FcRγ after Dectin-1 activation (Figure 8), although the indirect association could also be possible. In addition, we detected the binding of SHP-2 to FcRγ. However, SHP-2 has been reported to positively regulate Dectin-1-induced activation in response to fungal infection (28). Thus, the recruitment of SHP-2 may be irrelevant to the negative modulation of FcRγ here. In another study, the adaptor protein downstream of kinase 3 (DOK3) has been found to physically associate with the ITAM of DAP12 through its phosphotyrosine-binding domain and mediate the mitigation of LPS signaling in macrophages (41). It remains to be determined whether DOK3 is associated with FcRγ in Dectin-1 responses in DCs.

In this study, we reported for the first time that PTEN was recruited to FcRγ after Dectin-1 activation (Figure 8). Although we could not exclude the indirect binding of PTEN to FcRγ, PTEN has been identified as a negative regulator of FcR signaling, an ITAM-based signaling event. For examples, PTEN abrogates FcγR-mediated phagocytosis (42, 43), and the suppression of PTEN is critical for mast cell homeostasis and FcεRI-responsiveness (44). Notably, prostaglandin E2 (PGE2) enhances SHP-1 activity, resulting in increased PTEN activity. This mechanism contributes to the ability of PGE2 to abolish innate immune signaling in primary macrophages (45) and probably provides an explanation for our results (Figure 8). Serezani et al. report that PTEN is a key regulator in PGE2-mediated inhibition of phagocytosis of *C. albicans* (46), in agreement with our finding that FcRγ-associated PTEN might contribute to the negative regulation of antifungal responses. In addition, PTEN is responsible for the impairment of DC functions from elderly humans (47) and attenuates engulfment of apoptotic cells and apoptotic cell-induced anti-inflammatory response (48). It would be interesting to know whether PTEN-associated FcRγ also has such functions in DCs.

In the research of tumor immunology, Dectin-1 expressed on DCs and macrophages is critical to NK-mediated killing of tumor cells (12). Elucidating the molecular mechanism of Dectin-1-induced signaling in immune cells is essential for the design of new therapeutic strategies against cancer (49). Recently, soluble β-glucan has been reported to induce tumor regression in synergy with TLR9 agonist *via* DC-mediated immunity (50). In addition, curdlan has been reported to activate DCs through Dectin-1 and TLR4 signaling and the combination of curdlan and DCs efficiently inhibit tumor growth in mice (51). Our findings about the enhancement of TLR (22) and Dectin-1 (here) responses in FcRγ-deficient DCs may be applicable in promoting these treatments. Remarkably, Zhao et al. illustrate that immunization of tumor-bearing mice with Dectin-1-activated DCs induces potent antitumor response, due to Dectin-1-activated DCs as a powerful inducer of Th9 cells and antitumor immunity (52). It could be expected that the loss of FcRγ may facilitate Th9 differentiation by Dectin-1-activated DCs because of the enhanced Dectin-1 signaling.

In summary, we show here that FcRγ has inhibitory effects on Dectin-1 responses in DCs. Dectin-1 signaling is a key regulator of DC function in antifungal immune responses. In addition to antimicrobial host defense, Dectin-1 also plays roles in homeostasis, autoimmunity, allergy, and the recognition and response to dead and cancer cells (7). Our finding suggests that fungi and cancer cells could probably use such FcRγ-mediated negative regulation to escape the Dectin-1-induced immune responses. Thus, FcRγ could be an ideal target for strengthening the function of Dectin-1-activated DCs. Interestingly, it has been shown to improve the potency of a DC-based tumor vaccine with a small interfering RNA (siRNA) targeting PTEN (53). Therefore, removing the FcRγ-mediated inhibition of Dectin-1 signaling by siRNA, or by CRISPR technology (54), might promote immunity and potentially could be applied to vaccine development and cancer therapy.

## Ethics Statement

All mice were housed in the barrier facility in College of Medicine, National Taiwan University (Taiwan) under an Institutional Animal Care and Use Committee-approved protocol (Permit Number: 20110432). All animal experiments were performed in accordance with the Guide for the Experimental Animal Research Laboratory of the National Laboratory Animal Center, Taiwan. We did our best to minimize suffering during the animal experiments.

## Author Contributions

Y-GP, Y-LY, and C-CL performed experiments. LLL contributed materials and edited manuscript. C-LC designed experiments and prepared the manuscript.

## Conflict of Interest Statement

The authors declare that the research was conducted in the absence of any commercial or financial relationships that could be construed as a potential conflict of interest.
